# Bacterial Infections in Neonates, Madagascar, 2012–2014

**DOI:** 10.3201/eid2404.161977

**Published:** 2018-04

**Authors:** Bich-Tram Huynh, Elsa Kermorvant-Duchemin, Perlinot Herindrainy, Michael Padget, Feno Manitra Jacob Rakotoarimanana, Herisoa Feno, Elisoa Hariniaina-Ratsima, Tanjona Raheliarivao, Awa Ndir, Sophie Goyet, Patrice Piola, Frederique Randrianirina, Benoit Garin, Jean-Marc Collard, Didier Guillemot, Elisabeth Delarocque-Astagneau

**Affiliations:** Institut Pasteur, Paris, France (B.-T. Huynh, M. Padget, D. Guillemot, E. Delarocque-Astagneau);; Assistance Publique–Hôpitaux de Paris Hôpital Universitaire Necker-Enfants Malades and Université Paris Descartes, Paris (E. Kermorvant-Duchemin);; Institut Pasteur de Madagascar, Antananarivo, Madagascar (P. Herindrainy, F.M.J. Rakotoarimanana, H. Feno, E. Hariniaina-Ratsima, T. Raheliarivao, P. Piola, F. Randrianirina, B. Garin, J.-M. Collard);; Institut Pasteur de Dakar, Dakar, Senegal (A. Ndir);; Institut Pasteur du Cambodge, Phnom Penh, Cambodia (S. Goyet)

**Keywords:** bacterial infection, drug resistance, antimicrobial resistance, developing countries, low-income countries, infant, newborn, neonate, bacteria, Madagascar

## Abstract

Severe bacterial infections are a leading cause of death among neonates in low-income countries, which harbor several factors leading to emergence and spread of multidrug-resistant bacteria. Low-income countries should prioritize interventions to decrease neonatal infections; however, data are scarce, specifically from the community. To assess incidence, etiologies, and antimicrobial drug–resistance patterns of neonatal infections, during 2012–2014, we conducted a community-based prospective investigation of 981 newborns in rural and urban areas of Madagascar. The incidence of culture-confirmed severe neonatal infections was high: 17.7 cases/1,000 live births. Most (75%) occurred during the first week of life. The most common (81%) bacteria isolated were gram-negative. The incidence rate for multidrug-resistant neonatal infection was 7.7 cases/1,000 live births. In Madagascar, interventions to improve prevention, early diagnosis, and management of bacterial infections in neonates should be prioritized.

Most deaths of children <5 years of age (6.3 million in 2013) still occur in low-income countries; a leading cause is infectious disease (*1*). In these countries, deaths of neonates are particularly concerning; in 2013, there were 20 deaths/1,000 live births, 23% directly attributable to severe infections (*1*–*3*). Each year in low-income countries, 7 million possible (clinical signs with no bacteriological documentation) severe neonatal bacterial infections occur (*4*,*5*). In these countries, multiple factors lead to enhanced emergence and spread of drug-resistant bacteria (e.g., antimicrobial drug misuse, poor quality or counterfeit drugs, and substandard hygiene and living conditions) (*6*,*7*). This phenomenon involves gram-positive (*Staphylococcus aureus* and *Streptococcus pneumoniae*) and gram-negative (*Haemophilus influenzae*, *Enterobacteriaceae*) bacteria (*8*). These pathogens, especially those acquired in hospitals, are becoming increasingly resistant to multiple drugs; for most populations in these settings, the antimicrobial drugs required to treat these infections are not affordable (*9*).

Because few data on the burden of invasive bacterial infections and resistance patterns in low-income countries are available, we do not have an accurate picture of their true burden among the youngest children. Indeed, most studies of antimicrobial drug resistance in neonates were conducted >10 years ago. Data about antimicrobial drug resistance were sparse and often relied on few isolates; no clear conclusions have been made with regard to *Enterobacteriaceae* resistance to third-generation cephalosporins (6%–97% of infections) or methicillin resistance among *S. aureus* (0–67%) (*10*,*11*). Moreover, data regarding infections occurring in the community, which may differ from those in hospitalized persons, are especially lacking. To our knowledge, incidence rates for severe resistant infections in neonates have not been estimated (*10*,*11*).

In low-income countries, investment and mobilization to control neonatal infections and antimicrobial drug resistance remain extremely low. As long as the real burden of these events remains unknown, the scope for public health decision-making will be limited (*10*,*12*). Therefore, to assess incidence, etiologies, and antimicrobial drug–resistance patterns of neonatal infections, we conducted a prospective study of a cohort of 981 newborns enrolled at birth in rural and urban communities in Madagascar, one of the poorest countries in the world, where the mortality rate for neonates is high (*13*). 

## Methods

This study was part of the Bacterial Infections and Antimicrobial Drug Resistant Diseases among Young Children in Low-Income Countries (BIRDY project, http://www.birdyprogram.org). The BIRDY project investigates and responds to consequences of bacterial sepsis and antimicrobial drug resistance in children <2 years of age (protocol in Technical Appendix). The study was authorized by the Institut Pasteur in Paris and by the Ethics Committee in Madagascar. Informed consent was obtained for all participants.

### Study Areas and Study Population

**Figure 1:**
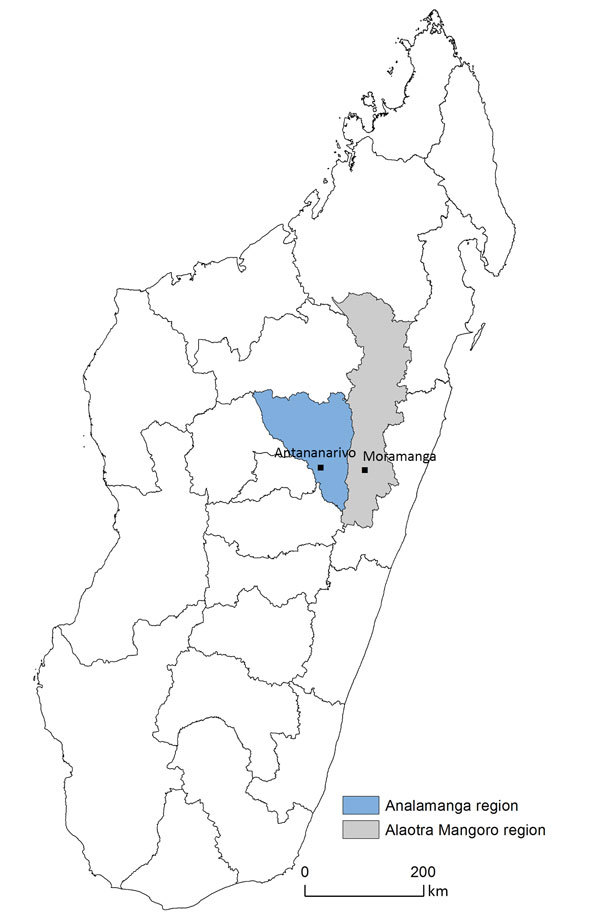
Locations of Antananarivo and Moramanga in Madagascar.

The study population included all neonates born in 3 districts (Avaradoha, Besarety, and Soavinadriana) of Antananarivo (the capital of Madagascar, with a catchment area population of 14,997 and 4,128 women of childbearing age) and those of the rural city of Moramanga (catchment area population of 17,159 and 3,795 women of childbearing age) (Figure 1). These areas were chosen because their populations, from poor to extremely poor, were representative of the general population. 

**Recruitment**

#### Before Birth

We exhaustively identified pregnant women within the study areas during their routine third trimester antenatal visit and pre-enrolled those who met the following criteria: routine residence in the study area with no plan to move away during the follow-up period and no opposition to the research being conducted or to the collection of biological samples (Technical Appendix). We actively monitored preincluded women to ensure enrollment of their neonates at birth. At the time of preinclusion or at delivery, a vaginal swab sample was collected from the pregnant women to detect group B *Streptococcus* (GBS).

#### At Birth

To ensure the exhaustiveness of live-birth recruitment, newborns were eligible at birth, even if their mothers had not been pre-enrolled. Neonate inclusion criteria were similar to preinclusion criteria of pregnant women: neonates born to parents living in the study area with no plan to move during the follow-up period; those whose legal guardians were informed and had no objection to the study procedures and collection of biological samples; and those for whom written consent was obtained from at least 1 legal guardian.

We collected fecal samples from the mothers perinatally to test for extended-spectrum β-lactamase (ESBL)–producing *Enterobacteriaceae*. We also collected the mothers’ sociodemographic, medical, and obstetric characteristics; delivery information; and the neonates’ anthropometric measurements and Apgar scores.

The neonates were examined at birth, and risk factors for infection (Technical Appendix) were assessed. The presence of risk factors for infection led immediately to collection of a placental biopsy sample and collection of gastric fluid (before the first feeding), deep auditory canal samples, and anal swab samples from the neonate to document perinatal bacterial colonization. We then referred neonates with suspected infection to a participating hospital for evaluation. When indicated, antimicrobial drugs were empirically administered according to the World Health Organization (WHO) criteria. For these neonates, we obtained blood samples and lumbar puncture samples (if indicated) beforehand (*14*).

### Follow-Up Evaluations

We actively and prospectively followed up on all neonates during their first month of life. To detect early signs of infection, we arranged for home visits to be conducted twice during the first week of life, beginning within 3 days after delivery. Routine checkups were then conducted weekly during the first month. We conducted active monitoring to minimize the number of missed or uncharacterized suspected infections and to obtain anthropometric measurements. Throughout follow-up, we asked mothers to contact an investigator whenever the child had a fever or showed signs suggestive of infection (Technical Appendix). If that occurred, the child was evaluated by a physician. When indicated, we collected samples including blood cultures according to the protocol and recorded clinical presentation, final diagnosis, and collected samples.

We adapted clinical criteria for infection and flow charts for bacterial sampling from WHO recommendations (Technical Appendix). Decisions regarding antimicrobial drug treatments were left to the attending physicians to decide according to local protocols.

### Bacteriology Analyses

All samples were transported within hours to Institut Pasteur in Madagascar for analysis. Specimen sampling, bacterial isolation, and species identification were performed according to the procedures recommended by the French Society for Microbiology (*15*). Antimicrobial susceptibilities were determined by use of the disk-diffusion method, according to the recommendations of the French Society for Microbiology (Technical Appendix) (*15*). Suspected ESBL-producing *Enterobacteriaciae* were confirmed by use of the double-disk synergy test. *Escherichia coli* ATCC 25922 was used for quality control strains.

### Classification Procedures

All cases for whom clinical or biological criteria for bacterial infection occurred during the neonatal period (including biological markers of infection based on C-reactive protein or complete blood count when available) were reviewed by an epidemiologist, a neonatologist, and a microbiologist to classify them and exclude nonsevere cases and contaminants. We defined severe bacterial infection as 1) presence of clinical signs of sepsis according to the WHO guidelines (Technical Appendix) and 2) a positive culture from blood or cerebrospinal fluid or urine (bacterial and leukocyte counts >10^5^ and 10^4^, respectively) or umbilical purulent discharge in case of omphalitis-associated sepsis. We defined 3 periods: very early (0–3 days), early (0–6 days), and late (7–30 days). We considered multidrug-resistant infections to be those caused by pathogens resistant to >1 agent in >3 antibacterial categories (*16*).

### Statistical Analyses

For our analyses, we used Stata version 12 (StataCorp, LLC, College Station, TX, USA). We used descriptive statistics (e.g., proportions, means, and SDs) to summarize characteristics of mothers and neonates. We compared differences in proportions and means by using the χ^2^ and Student *t* tests, respectively. p<0.05 was considered significant. We calculated the person-time (no. days followed until event [infection]) and then estimated the incidence of culture-confirmed severe neonatal infections per 1,000 live births. We calculated 95% CIs for all rates.

## Results

### Characteristics of Mothers and Neonates

**Figure 2:**
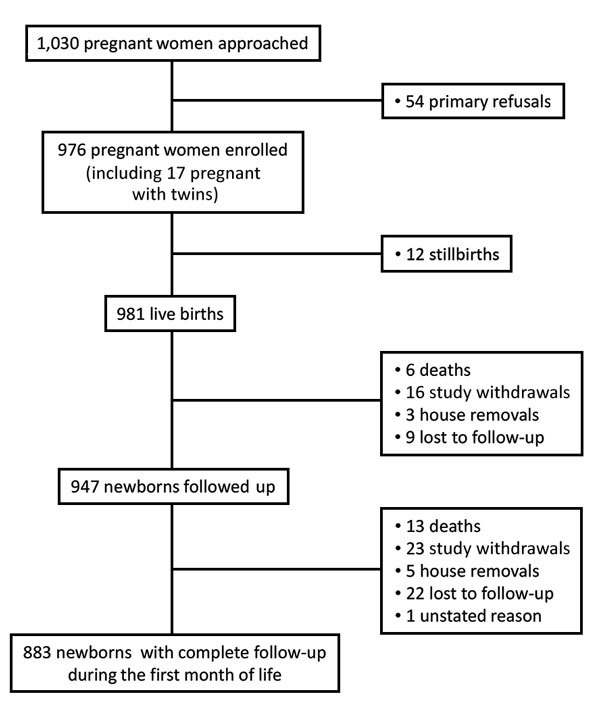
Flowchart for study of bacterial infections in neonates, Antananarivo and Moramanga, Madagascar, 2012–2014.

From September 2012 through October 2014, we approached 1,030 pregnant women, of whom 54 refused to be included and 976 were enrolled (Table 1; Figure 2); of those included, 393 (40.3%) were from the urban site and 583 (59.7%) from the rural site. On average, the women were 26.1 years of age (range 14–48 years of age) and 33.7% were primigravidae. A total of 351 (37%) women gave birth at home. At delivery, 981 live neonates were included; mean ± SD birth weight was 2,952.6 ± 504.4 g; of these neonates, 161 (16%) were premature (<37 weeks’ gestation).

**Table 1:** Characteristics of mothers and neonates enrolled in study of bacterial infections in neonates, Antananarivo and Moramanga, Madagascar, 2012–2014

Characteristic	Urban site, no. (%)	Rural site, no. (%)	p value
Pregnant women*†			
Parity			
Primigravida	144 (37)	185 (32)	>0.99
Multigravida	249 (63)	398 (68)	
Education			
None or primary	119 (30)	145 (25)	<0.001
Partial secondary	171 (44)	334 (57)	
Completed secondary or university	103 (26)	104 (18)	
No. antenatal visits at enrollment			
0–1	45 (11)	47 (8)	0.01
2–4	239 (61)	408 (70)	
>4	109 (28)	128 (22)	
Neonates‡			
M	215 (55)	277 (47)	0.03
F	179 (45)	310 (53)	
Premature, <37 wk of gestation	77 (19)	84 (14)	0.6
Risk factors for infection at delivery			
Fetid amniotic fluid	39 (9.9)	42 (7.1)	0.13
Prolonged membrane rupture	13 (3.3)	15 (2.6)	
Maternal fever at delivery	5 (1.2)	9 (1.5)	
Difficult birth	25 (6.3)	56 (9.5)	

*Mean (±SD, minimum–maximum) age of urban mothers 25.8 (6.7, 14.3–43.5) years and of rural mothers 26.2 (6.5, 14.3–48.1) years; p = 0.4. †Total = 976 pregnant women (393 urban and 583 rural), including 17 who had twin pregnancies and 12 who had stillbirths. ‡Total = 981 (394 urban and 587 rural). Mean (± SD) weights of 954 neonates at delivery were 2,921 (±515.9) g for urban and 2,973 (± 495.7) g for rural sites; p = 0.12.

### Incidence of Neonatal Infections

A total of 16 neonates were classified as having culture-confirmed severe infection (Technical Appendix). Of these, 12 (75%) infections occurred during the first week of life. The incidence rates were 17.7 (95% CI 10.8–28.9) culture-confirmed cases of severe neonatal infection and 13.3 (95% CI 7.5–23.4) culture-confirmed cases of early-onset severe neonatal infections per 1,000 live births. The incidence rates for culture-confirmed severe neonatal infections were 14.8 (95% CI 7.4–29.5)/1,000 live births in rural sites and 22.2 (95% CI 11.1–44.4)/1,000 live births in urban sites. The incidence rates for culture-confirmed severe neonatal infections were 15.6 (95% CI 7.0–34.6)/1,000 live births at home and 19.4 (95% CI 10.4–36.0)/1,000 live births at healthcare facilities. Final clinical diagnoses were sepsis for 13 and meningitis for 3 neonates.

### Samples and Pathogens 

We cultured 144 blood (including 65 [45.1%] at birth), 79 urine, and 7 cerebrospinal fluid samples from neonates with clinical signs of infection (Table 2). Among blood samples, results of 9 (6.3%) were positive and 8 (5.5%) others were considered to be contaminated. Among urine samples, results were positive for 39 (49.4%), of which 3 were associated with severe neonatal infection. One (14.3%) cerebrospinal fluid sample was culture-positive for *Pasteurella* spp., and 2 (28.6%) others grew gram-negative bacteria that could not be further identified. Gram-negative rods were detected in 13 (81.2%) samples from the 16 neonates with culture-confirmed severe infections; the most prevalent pathogen was *Klebsiella* spp.

**Table 2:** Pathogens isolated and characteristics of neonates with culture-confirmed severe infections, Antananarivo and Moramanga, Madagascar, 2012–2014*

Pathogen	No. isolates	Neonate age at infection, d				Sample type, no.	Place of birth			Premature			Site	
		0–3	4–6	7–13	14–30		Home	HCF		No	Yes		Urban	Rural
Gram-positive														
* Staphylococcus aureus*	1				1	Blood, 1	1				1		1	
* S. epidermidis*	1				1	Blood, 1	1				1		1	
* Streptococcus pneumoniae*	1				1	Blood, 1	1			1			1	
Gram-negative														
* Klebsisella pneumoniae*	4	1	3			Blood, 2; urine, 2	1	3		4			1	3
* K. oxytoca*	2	1	1			Blood, 1; urine, 1		2		2			2	
* Escherichia coli*	2	2				Blood, 2	1	1		1	1			2
* Enterobacter cloacae*	1	1				Blood, 1	1			1	1			1
* Acinetobacter baumanii*	1	1				Umbilical, 1		1		1				1
* Pasteurella *spp.	1				1	Spinal tap, 1		1		1				1
Gram-negative staining†	2	2				Spinal tap, 2		2		2	2			2

*HCF, healthcare facility. †Pathogens identified by staining as gram-negative but could not be cultured and, therefore, were not further identified.

### Antibacterial Resistance 

Among the 11 samples with gram-negative rods that could be tested for antimicrobial drug susceptibility, more than half showed resistance to cefotaxime (6/10) and more than one third were resistant to gentamicin (4/10) and ciprofloxacin (4/11) (Technical Appendix Table 2). Among the 14 isolates for which antimicrobial drug resistance data were available, 5 isolates were resistant to ciprofloxacin and 9 were resistant to co-trimoxazole. Of the 6 *Klebsiella* spp. isolates, 4 were ESBL producers. The isolated *Staphylococcus epidermidis* strain was resistant to methicillin.

 A total of 11 isolates were resistant to >1 antimicrobial drug of the combination recommended by WHO for cases of neonatal sepsis (ampicillin and gentamicin); 4 were resistant to both drugs. The incidence rates for severe neonatal infection resistant to 1 drug recommended by WHO was 7.7 (95% CI 3.7–16.2) cases/1,000 live births and to both drugs was 4.4 (95% CI 1.6–11.7) cases/1,000 live births. Seven isolates were multidrug resistant, and the incidence rate for multidrug-resistant severe neonatal infection was 7.7 (95% CI 3.7–16.2) cases/1,000 live births.

### Clinical Outcomes

In total, 19 neonates, including 2 sets of twins and 1 other twin, died during the follow-up period. Four died at home with no etiology documented, 3 deaths were the direct consequence of severe prematurity, 1 was caused by birth injury, and 1 was caused by neonatal tetanus. The 10 remaining infants who died showed clinical signs of severe infection; no blood cultures could be performed before death. Six neonates were premature. All deliveries took place in healthcare facilities, except for 1, which occurred at home. The mother of a pair of twins was positive for vaginal carriage of GBS. A total of 4 neonates received a combination of gentamicin and a third-generation cephalosporin, and 5 received penicillin in addition to the 2 other drugs. All neonatal deaths except 1 occurred in the first week of life. None of the 16 neonates with a culture-confirmed severe infection died.

## Discussion

Incidence of culture-confirmed severe neonatal infections in a community-based cohort of neonates in Madagascar was high (17.7 cases/1,000 live births). These infections are usually difficult to document, especially where women frequently deliver their babies at home, because neonates may show few symptoms before the infections progress rapidly. By using active community recruitment and follow-up, we were able to identify severe neonatal bacterial infections, including those with very early onset. Also, performing blood cultures before initiating antimicrobial drug therapy increased the likelihood of identifying a pathogen.

In low-income countries, incidence estimates for severe neonatal infections are few and the available data are heterogeneous (*10*). On the basis of community recruitment, Darmstadt et al. estimated an incidence rate of confirmed severe neonatal infection of 2.9 (95% CI 1.9–4.2)/1,000 live births almost 10 years ago in Bangladesh; this rate is much lower than the one we found (*17*). However, the findings of Darmstadt et al. may be underestimated because of delayed care seeking and a shorter active surveillance period.

Our incidence estimate is lower than the 44.8 early-onset infections/1,000 live births found by Turner et al. on the Thailand–Myanmar border; their estimate was based on a clinical definition of infections and was thus possibly overestimated (*18*). Also, our incidence risk (1.6%, 16/981) is lower than the pooled incidence risk for possible severe bacterial infection (7.6%) estimated by Seale et al. in a metaanalysis of 22 studies (*5*). However, the designs of the studies contributing to the Seale analysis varied widely. Our community-based study with pre-enrollment of pregnant women before delivery is likely to reflect a higher ascertainment.

The bacterial isolation rate in blood culture is low (≈10%) for infected neonates in high-income and low-income countries (*19*). Blood cultures require that trained staff collect these samples before any antimicrobial drug use is initiated. These practices are not routine in low-income countries, and some highly suspected infections could not be bacteriologically confirmed, even in the setting of our research protocol, which included continuous training. However elevated, the incidence rate of confirmed severe infections may therefore be underestimated in our study.

As a comparison, in 2008, the United States reported 0.77 early-onset infections/1,000 live births (*20*). Although most studies in high-resource settings focus on the early neonatal period and are not population based, our results clearly suggest a much higher burden of neonatal infections in Madagascar than in high-income countries.

Most (75%) neonatal infections occurred during the first week after birth, most during the first 3 days. This finding confirms that community-based active surveillance in the very early period of life is crucial for capturing infections in neonates (*4*). This result also points out the value of reinforcing interventions and research programs targeting the perinatal period.

Gram-negative bacteria were predominant; the most prevalent pathogen isolated was *Klebsiella* spp. In a review of studies reporting the etiology of serious bacterial infections in community settings, Zaidi et al. found that *Klebsiella* spp., *E. coli*, and *S. aureus* were the most prevalent bacteria isolated during the first week of life (*21*–*25*). In our study, *S. aureus* was not predominant. It is possible that healthcare workers caring for mothers and neonates in our study were more prone to use clean birth kits distributed by the BIRDY program and to follow good hygiene practices, potentially minimizing horizontal transmission of *S. aureus* to newborns.

The overall burden of GBS infection in the developing world is not clear; incidence ranges from 0.3 to 0.6 infections/1,000 live births (*26*). Our study identified no GBS infections. One possible explanation for this low incidence is that several early-onset GBS infections may have not been identified because of rapid death (*27*). However, this hypothesis is unlikely because we performed close and active surveillance directly after birth and no deaths occurred during the very short period between delivery and neonate enrollment. However, we cannot exclude the possibility that GBS might have been responsible for some cases of infection that could not be bacteriologically confirmed for neonates with clinical signs of sepsis, including some who died. In the context of GBS vaccine development, if confirmed, this low incidence may bring into question the potential cost-effectiveness of maternal vaccination in low-income countries.

We found that the proportion of multidrug-resistant infections was significant (50%, 7/14); 28.6% (4/14) of *Enterobacteriaceae* were ESBL producers, and 1 of the 2 *Staphylococcus* spp. isolates was resistant to methicillin. One striking result of our study, however, is the relatively low incidence of antimicrobial drug–resistant infections (≈7.7 infections/1,000 live births). We found no carbapenemase-producing *Enterobacteriaceae*. In most published studies, assessment of antimicrobial drug resistance at the community level is based on the proportion of resistant infections at hospital admission, which may lead to biased conclusions because of variability in care access and case severity. Our results enabled a more complete picture of this issue and suggest that multidrug-resistant infections in the community may be less problematic than previously estimated.

Nevertheless, more than three quarters (11/14) of the pathogens we isolated were resistant to at least 1 antimicrobial drug recommended by WHO, including 4 isolates resistant to both recommended drugs (*14*). These findings are consistent with those of several studies conducted in hospital or community settings, which also highlight reduced susceptibility to at least 1 antimicrobial drug recommended for empirical treatment (resistance ranging from 43% to 97%) (*19*,*22*,*28*). In contrast, we observed that the most frequent attitude of physicians in Madagascar was to prescribe 3 antimicrobial drugs, including ampicillin, a third-generation cephalosporin, and gentamicin, when invasive bacterial neonatal infection was suspected. The use of large and unnecessarily broad-spectrum therapy may contribute to increased rates of antimicrobial drug resistance. The development of rapid diagnostic tests to identify pathogens and their antimicrobial drug susceptibility may therefore prevent unnecessary use of broad-spectrum antimicrobial drugs.

Our study has some limitations. We cannot exclude the possibility that 4 pathogens, including one ESBL-producing *Enterobacteriaceae*, which we documented in the community, might have been acquired in the hospital because the infections occurred after 48 hours in neonates born in a healthcare facility. However, no hospitalization during pregnancy was recorded for the mothers of any of these 4 neonates. Also, because the neonates were enrolled in a research study, their standard of care might have been higher, including better hand hygiene, than that for most of the population. This Hawthorne effect might have induced bias in our results, such as an underestimation of *Staphylococcus*-associated infections (*29*). We also probably changed the evolution of these severe bacterial infections by improving early diagnosis and providing better care. These actions might have helped avoid deaths of neonates, which would otherwise have occurred, and contributed to our underestimation of case-fatality ratio.

In conclusion, incidence of bacterial infections among neonates in a community-based cohort in Madagascar was high, although incidence of multidrug-resistant bacterial infections was relatively low. Most of these infections occurred during the first week of life. Our findings suggest that public health measures to decrease deaths from severe bacterial infection among neonates should focus on improving prevention, early diagnosis, and management of infections and prioritizing intervention strategies according to successes with vaccines, clean deliveries, and care of neonates. Current knowledge gaps, including those associated with local burden, bacterial etiology, and antimicrobial drug resistance profiles of severe bacterial infections in low-income countries, prevent us from having a clear picture of the situation. Recently, several international bodies called for global action to combat antimicrobial drug resistance, deemed a “global health security threat” (*11*,*30*). Although antimicrobial drug resistance is a real threat, more community data are clearly needed in countries with limited resources so they can select and prioritize effective preventive and treatment strategies to tackle bacterial infections in neonates.

Technical Appendix Additional methods, neonate clinical signs, and results of antimicrobial susceptibility testing in study of bacterial infections in neonates, Madagascar, 2012–2014.
