# Supplementary material for: Bacterial Infections in Neonates, Madagascar, 2012–2014
Source: Emerg Infect Dis. 2018 Apr;24(4):710–7. doi: 10.3201/eid2404.161977 (PMC5875286; doi:10.3201/eid2404.161977)
Supplement: Technical Appendix — Additional methods, neonate clinical signs, and results of antimicrobial susceptibility testing in study of bacterial infections in neonates, Madagascar, 2012–2014. [file 16-1977-Techapp-s1.pdf]

Article DOI: https://doi.org/10.3201/eid2404.161977

Bacterial Infections in Neonates, Madagascar, 2012–2014

Technical Appendix

Section 1: Methodology of the BIRDY program

1. Development within a site

In each site, the program has two distinct phases. The first is **a pilot phase lasting approximately 1 year.** This phase aims at solving possible issues **regarding** data collection, organization, and logistics, before planning the full investigation, to begin during the second year.

Therefore, the specific objective of the pilot phase is to assess the feasibility of the study, at several sites, in real-life situations and to identify potential implementation problems and to find the solution to these problems.

During the pilot phase, we plan to recruit circa 1000 live born neonates at each site, combining both rural and urban setting.

After the pilot phase, the program continues into the complete program using the same geographic basis, with the aim of recruiting ≈1000 live births per year.

2. Study population and recruitment sources

The study population consists of neonates followed from birth until the age of 2 years. Two periods have been identified for recruitment: at birth, or before birth in the so–called “pre-inclusion” phase.

3. General description of the cohort

The general organization of the investigation is illustrated in Technical Appendix Figures 1 and 2.

3.1 Recruitment (see Technical Appendix Figure 1)

Pregnant women

At each site, we first organize the exhaustive identification of pregnant women within a geographically based defined population. Each pregnant woman is asked to participate in the study.

Pregnant women are systematically provided with information about the project during prenatal consultations.

Pre-inclusion occurs during third trimester consultation for all women meeting the pre-inclusion criteria (see Section 2) and giving informed written consent.

At the time of pre-inclusion, the "maternal" section of the electronic Clinical Report Form (e–CRFm) concerning the socio-demographic, medical, and obstetric characteristics of the mother is completed.

The neonates born from the pre-included mothers are included in the study at the time of delivery.

Given the main objective of the study, which relates to the measurement of the incidence of bacterial infections, and the potentially large number of women who do not have antenatal consultations, we optimize the exhaustiveness of live birth recruitment by also including neonates at the time of birth, without a prior pre-inclusion phase.

Births are much easier to identify for deliveries in healthcare structures participating in this project. However, because of a high proportion of home births, particularly in rural environments, we ensure that women giving birth outside healthcare structures are identified as rapidly as possible, through regular monitoring of mothers around the predicted date of delivery, by using text messages and mobile phones and by involving community workers, particularly in rural environments, and working with the matrons.

Investigators seek information daily from healthcare structures and matrons participating in the project concerning any births occurring over the last 24 hours.

To help ensure that the recruitment of the children is exhaustive, we also compare our data with the official birth registry where possible.

At birth

Neonates are included in the study if they meet the inclusion criteria (see Section 2).

A rectal and a vaginal swab sample is taken in mothers giving birth in a healthcare structure.

The e–CRFm is completed at the time of the delivery if the mother has not already been pre-included, and the neonate is thus recruited at the time of delivery.

At the time of birth, an initial "child" e–CRFc concerning the characteristics of the delivery is completed. Anthropometric measurements (weight, length, head circumference and brachial circumference) are recorded. An APGAR score is obtained by the care staff handling the delivery, one, five, and 10 minutes after delivery. The neonate is examined by a health care worker who checks the presence of risk factors for infection (Section 3).

If the mother gives birth with the assistance of a matron (not in a health care structure) the investigator is notified as soon as possible, for the collection of medical information from the matron concerning the birth and the neonate. The investigator checks the presence of risk factors for infection. Babies at risk for infection are referred to a participating hospital for pediatric evaluation. If the neonate dies before the arrival of the investigator, the e–CRFc is completed and a verbal autopsy is carried out by the investigator with the matron and, if possible, the mother or a relative.

The presence of a risk factor for infection at birth systematically leads to collection of the following samples within few hours after birth: gastric fluid (before the first feed), deep auditory canal swabs, anal swabs and a placental biopsy[1].

The decision as to whether to administer an empirical antibiotic treatment at birth is made in accordance with WHO criteria. If the decision is made to begin empirical antibiotic treatment, blood cultures and blood sampling for CRP determination is performed beforehand in addition to the collection of the systematic samples. A chest X- ray and lumbar puncture may also be requested, at the discretion of the clinician, according to the clinical context.

3.2 Follow–up (see Technical Appendix Figure 2)

General organization

After their births the children are followed for the first 2 years of their lives. Follow-up is both passive and active.

Throughout the follow–up period, passive follow–up consists in asking the mother to contact an investigator whenever the child has fever or meets criteria suggestive of infection (Section 3). An information leaflet describing these criteria is distributed and explained to the mothers beforehand. The mothers are also provided with a thermometer and asked to check the child's axillary temperature. Body temperature is monitored daily during the first month of life, and then weekly for the rest of the follow–up period. The temperatures are recorded on a paper document (which is adapted appropriately if the mother is unable to read).

Close active follow–up is also carried out to minimize the number of missed or uncharacterized infections. During the first 7 days of life, an update concerning the child is requested daily by sending a text message to a mobile telephone. In addition, two home visits are planned, the first one within 3 days of delivery. During this initial visit, the investigator determines the gestational age of the newborn using "Ballard" score. (The Ballard Maturational Assessment, Ballard Score, is a commonly used technique of gestational age assessment. This involves a clinical examination of the neonate evaluating physical and neurologic maturity. It assigns a score to various criteria, the sum of all of which is then extrapolated to the gestational age of the baby. These criteria are divided into Physical and Neurologic criteria. This scoring allows for the estimation of gestational age in the range of 26 weeks–40 weeks.)

Thereafter, routine check–ups take place weekly during the first month of life, then fortnightly until 3 months of age and monthly between 3 months and twelve months and then every 2 months between 1 and 2 years of age. These check-ups are carried out by investigators and makes it possible to note the occurrence of infectious episodes that have not already been identified, to detect possible infections at the time of the check-up, to remind the mother of the importance of the continuing participation of her child in the cohort, and to provide useful information for the follow–up of the child. The child is also weighed and measured (height, brachial circumference, and head circumference).

All the investigators participating in this project are trained in techniques for weighing the children, taking anthropometric measurements, determining “Ballard” scores and in the clinical evaluation of infection criteria. Furthermore, regular checks are made to ensure the correct execution of the various measurement techniques.

3.3 Presence of infection criteria during follow-up (see Technical Appendix Figure 3)

A criterion for infection may be detected by the mother, who may then consult at the hospital or the Primary Care Center (PCC), or call the investigator. The temperature chart completed by the mother is shown to the investigator during his or her visit, or to the staff of the health center or hospital in cases of direct consultation.

A criterion for infection may be observed by the investigator during routine check–up visits or during a visit at the request of the mother.

When fever is confirmed (axillary temperature $\geq$37.5C) or in the presence of other clinical criteria for infection, the child is to be examined by a medical doctor (at the reference/district hospital or at the PCC). If the investigator has followed initial basic paramedical or medical training, he or she may determine whether the state of health of the child requires immediate hospitalization or whether the treatment at the PCC is possible. If the investigator has no basic paramedical or medical training, he or she sends the child directly to hospital if a criterion of infection is detected.

The signs and symptoms of the child, the final diagnosis and the samples taken are recorded in the e–CRFc.

The medical evaluation is carried out by the attending medical doctor, who completes a paper questionnaire. At the time of data entry, the investigator checks that the collected information is complete, and adds any missing information, with the assistance of the doctor, when necessary:

- Systematic samples in cases in which clinical criteria for suspected infection are identified: urine samples for cytobacteriological examination; blood for blood cultures; lumbar puncture in febrile children under the age of 3 months; and thick blood smears if the child has a

fever in a malaria-endemic area. Blood formula and C–reactive protein (CRP) determinations are also carried out.

- Samples based on the presence of particular warning signs of infection: stool samples for coproculture in cases of diarrhea (at least three liquid stools per day); lumbar puncture in the presence of neurologic signs or convulsions; swabbing in cases of discharge from the eyes or ears or in cases of signs of omphalitis (swabbing of the pus).

When possible, a chest x-ray is carried out in the presence of respiratory signs, at the discretion of the attending clinician. Samples for bacteriological analysis are transported as rapidly as possible in a cold box encased in secure packaging to the microbiological laboratory of the Institut Pasteur of each site.

Section 2: Criteria for inclusion and exclusion

Preinclusion of the women during pregnancy

Preinclusion criteria:

- Routine residence in the study zone of a participating country
- No plans to move away from the study zone during the period of follow-up for the neonate
- Information provided about the way in which the study will be carried out and about the collection of biologic samples from the neonate
- No opposition from the pregnant woman to the research being carried out or to the collection of biologic samples
- Signed informed consent form.

Exclusion criteria:

- Residence outside the study zone of a participating country
- Plans to move away from the study zone during the follow-up period of the neonate
- No information provided about the way in which the study is carried out or about the collection of biologic samples from the neonate

- Opposition from the woman to the research being carried out or to biologic samples being collected from the neonate.

Inclusion of the neonate at delivery

Inclusion criteria:

- Neonate born to parents living in the study zone of a participating country
- Parents of the neonate not intending to move away from the study zone during the follow-up period
- Legal guardians of the neonate informed about the way in which the study is to be carried out and about the collection of biologic samples
- Legal guardians of the neonate having no objection to the collection of biologic samples
- Authorization from at least one of the legal guardians of the child, in the form of a signed informed consent form.

Exclusion criteria:

- Stillborn neonate
- Parents of the neonate living outside the study zone of a participating country
- Neonate born to parents planning to move away from the study zone of a participating country during the follow-up period
- At least one of the legal guardians of the neonate not informed about the study or about the collection of biologic samples
- At least one of the legal guardians of the neonate opposed to the collection of biologic samples.
- Neonate already participating in another biomedical study.

NB: being below the age of majority is not considered a criterion for the non-preinclusion of a mother or her infant. However, in such cases, informed consent must be obtained from the mother herself and from one of her parents or legal guardians.

Section 3A: Screening criteria for suspected infection

At birth

Risk factors for infection at birth leading to perinatal sampling and medical evaluation (risk factors are checked by the matron or the midwife in case of home birth and by the midwife or the attending doctor in case of hospital birth):

- Unplanned preterm delivery (<37 weeks gestation)
- Prolonged membrane rupture (≥12 h)
- Maternal fever (axillary temperature >37.9 C) at the time of delivery
- Low birthweight (<2500 g)
- Difficult birth (birth asphyxia)
- Foul-smelling amniotic fluid
- Infection in a twin
- Leukorrhoea or untreated urinary infection during pregnancy
- Home birth

During the neonatal period (0–28 days):

Criteria for suspected infection leading to medical evaluation and bacterial sampling (criteria used by healthcare agents):

- Feeding difficulties
- Restlessness, irritability
- Lethargy, movement only when stimulated, hypotonia, coma
- Bulging fontanelle
- Convulsions
- Abdominal distension
- Paleness or gray skin

- Redness around umbilicus or purulent discharge from the umbilicus
- Prolonged capillary refill (>3s)
- Respiratory rate >60/min
- Apnoea (>15s) or bradypnoea (respiratory rate <20/min)
- Difficult breathing (grunting or severe chest indrawing)
- Cyanosis
- Hypothermia (<35.5C)
- Fever (axillary temperature >37.5C)
- Purulent discharge from the eyes
- Marked jaundice
- Many or severe skin pustules

Section 3 B: Criteria of infection (adapted from the WHO criteria)

During the neonatal period (0–28 days):

- Feeding difficulties
- Restlessness, irritability
- Lethargy, movement only when stimulated, hypotonia, coma
- Bulging fontanelle
- Convulsions
- Abdominal distension
- Paleness or gray skin
- Redness around umbilicus or purulent discharge from the umbilicus
- Prolonged capillary refill (>3s)
- Respiratory rate >60/min

- Apnoea (>15s) or bradypnoea (respiratory rate <20/min)

- Difficult breathing (grunting or severe chest indrawing)

- Cyanosis

- Hypothermia (<35.5C)

- Fever (axillary temperature >37.5C)

- Purulent discharge from the eyes

- Hepato- or splenomegaly

- Abnormal pulmonary auscultation

- Marked jaundice

- Many or severe skin pustules

Only severe bacterial infections were considered to evaluate incidence rates. Severe bacterial infections were defined as meningitis, pneumonia, or sepsis, irrespective of its etiology, based on the presence of clinical signs predicting severe bacterial illness from the WHO Young Infants Clinical Signs Study (in bold characters) (Young Infants Clinical Signs Study Group. Clinical signs that predict severe illness in children under age 2 months: a multicenter study. Lancet 2008; 371: 135–42).

After the age of 28 days:

- Changes in behavior

- Feeding difficulties

- Restlessness, irritability

- Lethargy, movement only when stimulated, hypotonia, coma

- Bulging fontanelle

- Convulsions

- Abdominal distension

- Paleness or gray skin

- Prolonged capillary refill (>3s)

- Respiratory rate >60/min

- Apnoea (>15s) or bradypnoea (respiratory rate <20/min)

- Difficult breathing (grunting or severe chest indrawing)

- Cyanosis

- Abnormal pulmonary auscultation

- Hypothermia (<35.5C)

- Fever (axillary temperature >37.5C)

- Marked jaundice

- Purulent ear discharge

- Purulent eye discharge

- Redness around umbilicus or purulent discharge from the umbilicus

- Painful, swollen joints

- Diarrhea, vomiting

- Hepato- or splenomegaly

- Poor weight gain (unless isolated)

- Many or severe skin pustules, skin rash

Criteria for empirical antibiotic treatment at birth[7]

- Presence of one or several clinical signs consistent with infection (see above (*3*))

- If the child is asymptomatic during the neonatal period: signs suggestive of chorioamnionitis (at least two of the following signs: prolonged membrane rupture (≥24 h); maternal fever (>37.9C) at the time of delivery; foul-smelling amniotic fluid) or infection in a twin

Section 4: Microbiological procedures

Blood cultures

Incubation was done at 35 ± 2°C up to 5 days if an automated device was used or 7 days if a manual procedure was applied. In case of manual procedure and use of a two-phase impregnation bottle, a daily inspection supported by the spread of liquid medium on a solid medium were carried out.

If positive, a direct microscopic examination of the broth and Gram staining were performed.

Cultures

Inoculation of broth and /or colonies on fresh blood agar and chocolate media under a 10% CO_2 atmosphere at 35 ± 2°C for 24 hours was done.

If multi-microbial growth occurred, then selective media were used.

Identification of isolated colonies was made with API galleries. If *Staphylococcus aureus* was suspected, identification was performed by coagulase and agglutination tests (Pastorex®). Susceptibility testing was done according to CASFM guidelines.

Screening of microorganisms

Screened bacteria were *Haemophilus influenzae, Streptococcus pneumoniae* and *agalactiae*, *Enterococcus* spp., *Staphylococcus aureus* and coagulase negative, *Neisseria meningitidis*, Enterobacteriacea and, non-enterobacteria gram negative bacilli.

Urine cyto-bacteriology tests

Macroscopic examination is followed by microscopic examination.

A leukocyte count (/mL) with a Malassez cell (or Nageotte) on homogenized urine (threshold 10^4/mL) was performed followed by a Gram staining on unspun urine.

Cultures

Inoculation on selective media was calibrated with a loop of 10µl. Culture media were incubated at 35 ± 2°C for 24 hours (optionally 48 h).

Identification of isolated colonies was made with API galleries. If *Staphylococcus aureus* was suspected, identification was performed by coagulase and agglutination tests (Pastorex®). Susceptibility testing was done according to CASFM guidelines.

Screening of microorganisms

Screened bacteria were *Escherichia coli*, *Proteus* spp., *Klebsiella* spp., *Enterobacter* spp., *Citrobacter* spp., *Pseudomonas aeruginosa*, *Acinetobacter* spp., *Enterococcus* spp., *Streptococcus agalactiae*, *Staphylococcus aureus* and *saprophyticus*, *Candida* spp.
Urine specimens were stored at +4°C until the release of results.

Cerebral spinal fluid cultures

Macroscopic examination classified CSF samples in clear, hemorrhagic, cloudy or citrine.

CSF microscopic examination

Erythrocyte/leukocyte counts were performed after homogenization with a counting chamber (Malassez). Quantitative cytology was done after cytocentrifugation, and Gram staining allowed tovisualize the microbial flora.

Soluble antigen detection included *Haemophilus influenzae type B*, *Streptococcus pneumoniae*, *Neisseria meningitidis* (serogroups A, C and W/Y), *Streptococcus agalactiae* and *Escherichia coli* K1.

Cultures

Two drops of CSF were spread on a chocolate agar supplemented with polyvitex and a blood agar medium and were inoculated into a Brain-Heart-Infusion (BHI) broth. These inoculated media were incubated at 36 ± 1°C with 10% CO_2 (except BHI) for 5 days.

Identification of isolated colonies was made with API galleries. If *Staphylococcus aureus* was suspected, identification was performed by coagulase and agglutination tests (Pastorex®). Susceptibility testing was done according to CASFM guidelines.

Screening of microorganisms

Screened bacteria were *Haemophilus influenzae*, *Streptococcus pneumoniae* and *agalactiae*, *Neisseria meningitidis*, *Escherichia coli* K1, *Klebsiella* spp., *Listeria monocytogenes* (aerobic incubation), *Staphylococcus* spp.

Culture-negative CSF with strong suspicion of meningitis were stored at −80°C.

Stools

A calibrated amount of stools was undertaken at baseline and reproduced throughout the project so as to use semiquantitative methods in bacteria counts.
Microscopic examination included direct examination and examination after staining with methylene blue and Gram.

Cultures

Some media were used systematically, Bromo-Cresol-Purple (BCP), Eosin-Methylene-Blue (EMB), Hektoen, Mueller-Kaufmann broth to screen for pathogenic enterobacteria, mainly *Escherichia coli*, *Salmonella* and *Shigella*.
In case of a bloody stool, a Cefsulodin-Irgasan-Novobiocin (CIN) agar was added.
Based on microscopic examination observation, additional solid media could be used, Blood agar supplemented with nalidixic acid for *Staphylococcus.aureus*, Thiosulfate-Citrate-Bile-Saccharose (TCBS) for *Vibrio* spp., and Chromagar Candida for yeast detection.
Media were incubated at 35 ± 2°C (except CIN) for 24 to 48 hours.
Identification of isolated colonies was made with API galleries. If *Staphylococcus aureus* was suspected, identification was performed by coagulase and agglutination tests (Pastorex®).
Susceptibility testing was done according to CASFM guidelines.

Serotyping

Salmonella were serotyped according to the Kaufmann-White scheme, *E. coli* O157:H7 and Shigella were serotyped with the appropriate antiserums according to the identified species.

Pus

Macroscopic examination was followed by microscopic examination which includes Gram staining to detect polymorphonuclear leukocytes and to examine the composition of bacterial flora.

Cultures

Pus were spread on chocolate agar, blood agar, Chapman and BCP.
In case of anaerobic suspicion, Schaedler agar and broth were inoculated. Incubation was carried

out at 35 ± 2°C with or without CO_2.

Identification of isolated colonies was made by API galleries. If *Staphylococcus aureus* was suspected, identification was performed by coagulase and agglutination tests (Pastorex®). Susceptibility testing was done according to CASFM guidelines.

Screening of microorganisms

Screened bacteria were *Staphylococcus aureus*, *Streptococcus β-hemolytic*, *Pseudomonas aeruginosa*, *Escherichia coli*, *Klebsiella* spp., *Enterobacter* spp., Serratia spp., *Acinetobacter* spp., *Alcaligenes faecalis*, *Stenotrophomonas maltophilia*, *Neisseria gonorrhoeae*.

Perinatal samples (placental, gastric fluid, deep auditory canal swabs, rectal swab)

Microscopic examination by Gram stain.

Cultures

Samples were inoculated on Blood Agar, chocolate Agar supplemented with Polyvitex (incubated under 10% CO_2) and on enterobacteria media when influenced by Gram staining observation. Monomorphic colonies were identified. Identification of isolated colonies was made with API galleries. If *Staphylococcus aureus* is suspected, identification was performed by coagulase and agglutination tests (Pastorex®). Susceptibility testing was done according to CASFM guidelines.

Screening of microorganisms

Screened bacteria were Enterobacteriaceae, *Staphylococcus aureus*, β-hemolytic streptococci, *Enterococcus* spp., *Pseudomonas aeruginosa*, *Acinetobacter* spp., *Listeria monocytogenes*

Technical Appendix Table 1. Clinical signs presented by 16 neonates with culture confirmed severe bacterial infection, Antananarivo and Moramanga, Madagascar, 2012–2014

Clinical signs	No. (%)
Hypothermia (<35.5°C)	3 (19)
Fever (axillary temperature >37.5°C)	7(74)
Feeding difficulties	4 (25)
Restlessness, irritability	7 (44)
Lethargy, movement only when stimulated, hypotonia, coma	6 (38)
Bulging fontanelle	1(6)
Paleness or gray skin	3 (19)
Redness around umbilicus or purulent discharge from the umbilicus	5 (31)
Prolonged capillary refill (>3s)	3 (19)
Respiratory rate >60/min	3(19)
Difficult breathing (grunting or severe chest indrawing)	3 (19)

Clinical signs	No. (%)
Cyanosis	2 (12)
Marked jaundice	4 (25)
Many or severe skin pustules	1(6)

Technical Appendix Table 2. Antimicrobial drug susceptibility of pathogens isolated from neonates with severe culture-confirmed infections, Antananarivo and Moramanga , Madagascar, 2012–2014*

Pathogen	AMP	AMC	TIC	GEN	AMK	TZP	TMP/ SXT	CEF	FOX	CTX	CAZ	CIP	ERY	IPM	CHL	TET	OXA	VAN	TEC
**Gram-positive**																			
Staphylococcus aureus				1/0			1/0		1/0			1/0			1/0	1/0		1/0	1/0
Staphylococcus epidermidis				1/1			1/1		1/1			1/0	1/0		1/1	1/1			1/0
Streptococcus pneumoniae	1/0			1/1			1/0					1/0	1/0		1/0	1/1	1/0	1/0	1/0
**Gram-negative**																			
Klebsiella pneumoniae	4/4	4/3		4/3	4/0	3/0	4/4	4/3	4/0	4/3		4/2		4/0	4/2				
Klebsiella oxytoca	2/2	2/2	1/1	2/1	2/0	2/0	2/2	2/2	2/1	2/2		2/1		2/0	2/2				
Escherichia coli	2/2	2/0		2/0	2/0	1/0	1/1	2/0	2/0	2/0		2/0		2/0	2/0				
Enterobacter cloacae	1/1	1/1	1/1	1/0	1/0	1/0	1/0	1/1	1/1	1/1		1/1		1/0	1/0				
Acinetobacter baumanii		1/0†	1/0	1/0			1/1				1/0	1/0	1/0	1/0	1/1				
Pasteurella spp.				1/0‡						1/0		1/0				1/0			

*AMC, amoxicillin/clavulanate; AMK, amikacin; AMP, ampicillin; CAZ, ceftazidime; CEF, cephalothin; cefalotin; CHL, chloramphenicol; CIP, ciprofloxacin; CTX, cefotaxime; ERY, erythromycin; FOX, cefoxitin; GEN, gentamicin; IPM, imipenem; OXA, oxacillin; TEC, teicoplanin; TET, tetracycline; TIC, ticarcillin; TMP/SXT, cotrimoxaxole; TZP, piperacillin/tazobactam; VAN, vancomycin. Data expressed as total no. isolates/no. resistant isolates.

†Ticarcillin/clavulanate.

‡Spectinomycin.

Technical Appendix Figure 1. Recruitment steps.

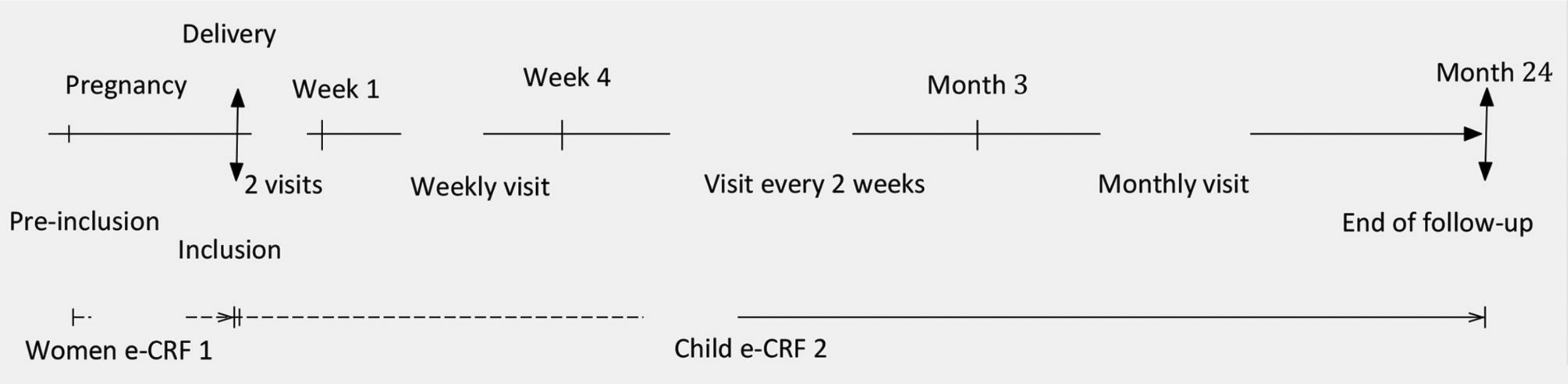

Technical Appendix Figure 2. Follow-up.

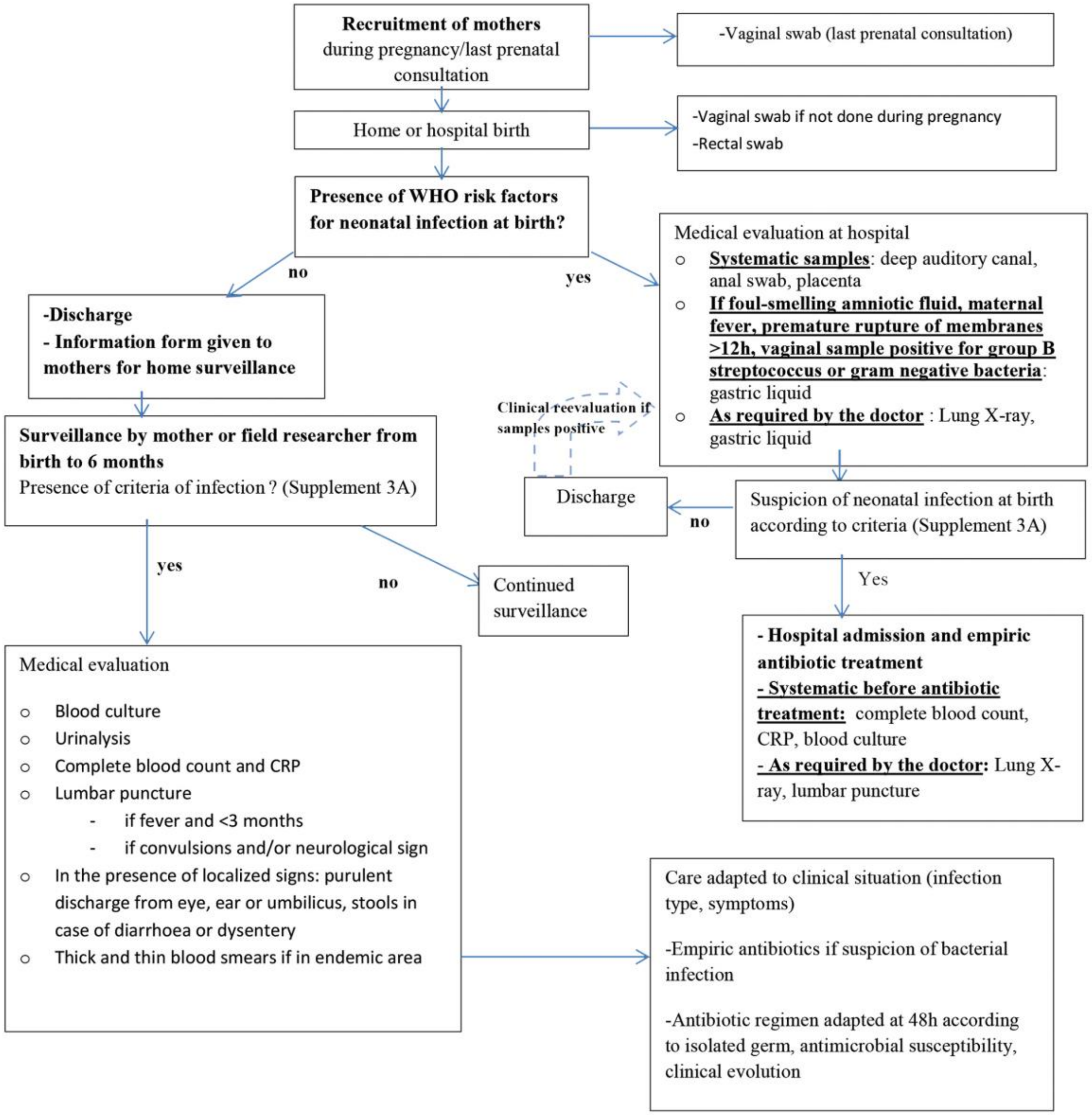

Technical Appendix Figure 3. Flow-chart for diagnosis and care of infants and newborns.
